# Fecundity compensation is dependent on the generalized stress response in a nematode host

**DOI:** 10.1002/ece3.5704

**Published:** 2019-10-01

**Authors:** Victoria L. Pike, Suzanne A. Ford, Kayla C. King, Charlotte Rafaluk‐Mohr

**Affiliations:** ^1^ Department of Zoology University of Oxford Oxford UK

**Keywords:** fecundity compensation, host–parasite interactions, indirect defense, life‐history shift, stress, trade‐off

## Abstract

**Background:**

Fecundity compensation, increased offspring output following parasite exposure, is widely reported, but the underlying mechanisms remain unclear. General stress responses are linked to other indirect defenses against parasites, and therefore may be responsible. We challenged strains of *Caenorhabditis elegans* (wild type and mutants with compromised or strengthened stress responses) with *Staphylococcus aureus*.

**Results:**

In all strains except the compromised stress response mutant, we saw elevated offspring production if hosts survived initial parasite exposure.

**Conclusion:**

We infer that general stress responses are linked with fecundity compensation. These results may explain why trade‐offs are not always observed among parasite defense mechanisms.

## Graphical abstract

We challenged strains of *Caenorhabditis elegans* (wild type and mutants with compromised or strengthened stress responses) with *Staphylococcus aureus*. We saw elevated offspring production if hosts survived initial parasite attack in all strains except the compromised stress response mutant.

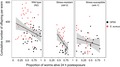

## 1 INTRODUCTION

Hosts can respond in many ways to parasite attack. They can launch an immune response to directly kill infecting parasites (Hoffmann, Reichhart, & Hetru, 1996), and modify their behavior either to passively avoid contact or to increase the probability of offspring surviving (Schaller & Park, 2011). A defense widespread across the animal kingdom is fecundity compensation (Heins & Baker, 2014; Minchella & Loverde, 1981; Schwanz, 2008; Thornhill, Jones, & Kusel, 1986; Vale & Little, 2012), whereby hosts increase offspring production upon exposure to parasites. This response can alleviate some of the fitness consequences of infection (Minchella & Loverde, 1981; Vale & Little, 2012). Fecundity compensation can occur either in the form of increased reproduction in early life (Minchella & Loverde, 1981) or whereby lifelong fecundity is increased (Vale & Little, 2012). Here, we use the term to refer to an increase in overall reproductive output in surviving individuals in the face of parasite attack (Vale & Little, 2012).

The mechanisms underlying fecundity compensation remain unknown. However, previous studies have suggested that indirect defenses against parasites are genetically linked to stress response pathways with involvement in the innate immune system (Schulenburg & Ewbank, 2007; Schulenburg & Müller, 2004). These indirect defenses, including behavioral avoidance, may be upregulated alongside physiological responses as part of a larger stress response to parasite exposure (Schulenburg & Ewbank, 2007). Thus, a hypothesis is that fecundity compensation is also part of this generalized response and dependent on genes linked to innate immunity.

Here, to investigate the link between stress and fecundity compensation, we exposed wild‐type and mutant nematode hosts with a heightened (IGF‐1 pathway mutant *daf‐2*) or suppressed (p38 MAPK mutant *sek‐1*) stress responses to *Staphylococcus aureus*. We then tracked the reproductive output of surviving hosts over time.

## 2 METHODS

To test whether the host generalized stress response could underlie the fecundity compensation mechanism, we used the model host, *Caenorhabditis elegans*. We exposed strains of *C. elegans* nematodes—wild type N2, IGF‐1 pathway mutant *daf‐2* (strain CB1368 daf‐2(e1368)) with heightened stress responses, and p38 MAPK mutant *sek‐1* (strain AU1 sek‐1(ag1)) with suppressed immunity and stress responses—to the opportunistic parasite *S. aureus* MSSA476. We then examined whether the nematodes that survived initial parasite exposure produced more offspring.

Host mortality and offspring production were measured following parasite exposure. Nematodes were chunked from the same ancestral plate to start each trial. Newly transferred worms were maintained at 20°C for 3 days before being surface‐sterilized (bleached) with sodium hypochlorite (Schulte, Makus, Hasert, Michiels, & Schulenburg, 2010) and synchronized in M9 overnight. Synchronized L1 nematodes were then plated onto 9‐cm petri dishes containing nematode growth medium (NGM) agar with a lawn of *E. coli* (80 μl grown overnight at 30°C in LB) and incubated at 20°C for 2 days. Subsequently, nematodes were removed from each plate and gravity‐washed using M9 containing 0.1% Triton‐X. Nematodes were then transferred to tryptic soy broth (TSB) agar cultured with lawns of *S. aureus* (80 μl grown overnight at 30°C in THB), and controls were also transferred to TSB agar but cultured with lawns of *E. coli* (80 μl grown overnight at 30°C in LB).

**Figure 1:**
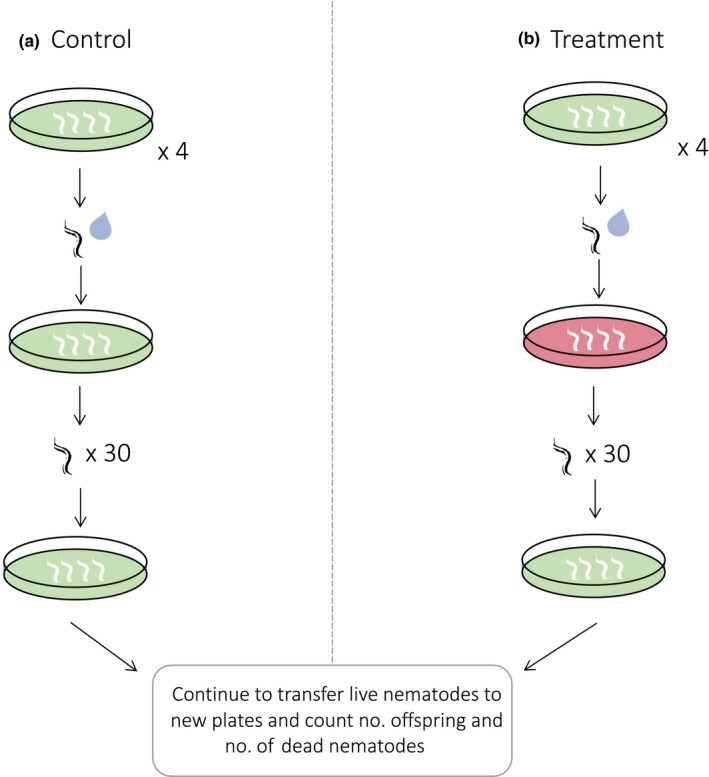
Experimental regime: synchronized L1 nematodes on NGM plate agar with a lawn of *E. coli* food (green) incubated at 20°C for 2 days. Nematodes were then washed using M9 containing 0.1% Triton‐X and transferred to lawns of *S. aureus* (red) or *E. coli* (control) on TSB agar. Thirty parent nematodes that survived the exposure to *S. aureus* (or were part of the control population) were picked using a platinum wire from each individual plate and transferred to new NGM plates seeded with food after 12 hr, 24 hr, and subsequently every 24 hr. The experiment continued until all of the nematodes were dead. Treatments consisted of four biological replicates, and the whole experiment was replicated five times

Thirty nematodes that survived the exposure to *S. aureus* (or were part of the control population) were picked using a platinum wire from each individual plate and transferred to new NGM plates seeded with *E. coli* after 12 hr, 24 hr, and subsequently every 24 hr. The transfer regime was selected after pilot experiments showed a peak in reproduction between 12 and 24 hr. After live nematodes were transferred, F1 progeny were washed off the plate using 2 ml of M9 with Triton‐X into a sterile 15‐ml falcon tube. The numbers of alive and dead nematodes were recorded prior to each transfer. The nematode mixture was then thoroughly vortexed, and the number of progeny present in each of the L1, L2, and L3 larval stages was counted in three aliquots of 5 μl, to provide replication and an accurate estimate of total number of F1 progeny. The numbers of live and dead nematodes were recorded after 24 hr. Dead nematodes were classified as those that did not respond via movement to being touched by platinum wire. The experiment continued until all of the nematodes were dead. Treatments consisted of four biological replicates, and the whole experiment was replicated five times (see Figure 1).

Data were analyzed in R version 3.2.0 and RStudio version 0.98.1091 (R Core Team, 2014). Differences in host mortality among strains at 24 hr after exposure to the parasite or control were analyzed using binomial general linearized models (GLMs) followed by Tukey's multiple comparison tests using the “multcomp” package in R. Overall differences in offspring production among strains were analyzed using ANOVAs followed by pairwise *t* tests with FDR *p*‐value correction. Spearman's rank correlations were used to examine the relationship between proportion of nematodes alive after 24 hr of parasite exposure and cumulative number of offspring over the course of the experiment. One‐way analysis of variance (ANOVA) tests were used to compare differences in offspring production following exposure to *S. aureus* or OP50 among nematode strains and specific differences analyzed using FDR‐corrected pairwise *t* tests.

## 3 RESULTS

Host survival following 24 hr of parasite exposure differed significantly across and between the host strains (binomial GLM: *χ* ^2^ = 134.4, *df *= 2, *p* < .0001; Tukey's tests: *p* < .001). The highest survival was seen in daf‐2 mutants (mean proportion survival of 0.6 ± 0.017 *SE*), followed by N2 (mean proportion survival of 0.46 ± 0.028 *SE*), and finally sek‐1 mutants (mean proportion survival of 0.27 ± 0.42 *SE*).

The overall number of offspring produced across treatments differed significantly among host strains both on OP50 food (ANOVA: *F* = 20.321, *df *= 2.77, *p* < .0001) and on *S. aureus* (ANOVA: *F* = 14.991, *df* = 2.77, *p* < .0001). This effect was entirely driven by the higher offspring production of N2 laboratory strain in both treatments (pairwise *t* tests: *p* < .0001), with N2 producing 248.38 ± 13.78 *SE* offspring on OP50 compared to 117.54 ± 8.27 *SE* for daf‐2 and 178.44 ± 18.49 for sek‐1 and N2 producing 374.40 ± 19.20 on *S. aureus* compared to 272.52 ± 13.43 for *daf‐2* and 224.17 ± 24.74 for *sek‐1*. Survival of control, unexposed hosts also differed significantly among host strains (binomial GLM: *χ* ^2^ = 153.32, *df* = 2, *p* < .0001). Compromised stress response *sek‐1* mutants survived less well on OP50 food with a mean proportion of 0.42 ± 0.05 *SE* survival after 24 hr compared to 0.72 ± 0.03 *SE* on N2 (Tukey's tests: *p* < .0001) and 0.68 ± 0.024 on *daf‐2*. There was no significant difference between N2 wild‐type nematodes and *daf‐2* mutants (Tukey's tests: *p* = .29). Life spans we report here are lower than those previously recorded for these strains grown on OP50. This is likely to be due to the stress from transferring steps. This full range of mortality values allows us to fully investigate the stress response, by exploring the correlation between harm (regardless of the source) and offspring production.

**Figure 2:**
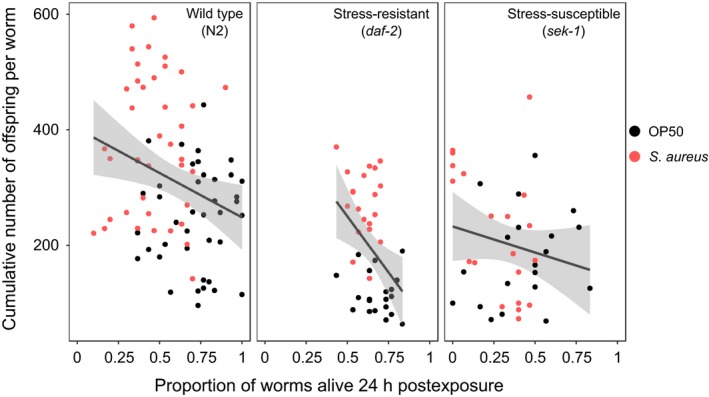
Relationship between host mortality after 24 hr of exposure (to parasites, red; to *E. coli* food, black) and cumulative number of offspring per nematode over lifetime for the three strains. Black lines represent linear regressions with gray areas showing 95% confidence intervals. Left to right from top: N2 wild type, daf‐2 mutant, and sek‐1 mutant

**Figure 3:**
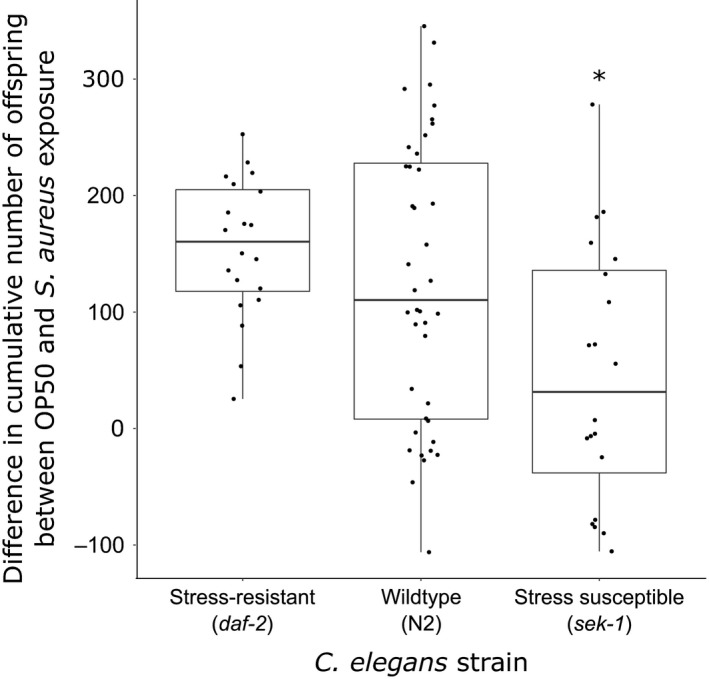
Difference in offspring production after host exposure to parasites and food. Strain with asterisk differs significantly from the wild type (N2; *p* < .05 pairwise *t* tests, FDR‐corrected). Individual data points are shown within boxplots

Of nematode hosts that survived parasite exposure, all host strains, except nematodes with suppressed stress responses, exhibited fecundity compensation, with *daf‐2* mutants having the most elevated response (Figure 2). Population‐level survival and cumulative offspring production per nematode were significantly negatively correlated (wild type [N2]: *S* = 109,040, *p* = .01, stress‐resistant strain: *S* = 14,991, *p*‐value = .0093; Figure 2). Thus, within strain type, the more the host population suffers from parasite exposure, the greater the fecundity compensation response by surviving hosts. Although the correlations were significant for N2 and *daf‐2*, the *R* ^2^ values for each are relatively low (*R* ^2^ for *daf‐2* = .18 and for N2 = .07), indicating that although the correlations were significant, there is a relatively high level of variation. Conversely, fecundity compensation was not demonstrated by nematodes with a compromised stress response, as no correlation between survival and overall fecundity was observed (*S* = 12,314, *p* = .3391; Figure 2). The extent of the response differed significantly among nematode strains (ANOVA: *F* = 5.8, *df *= 2.77, *p* = .004), and we observed the strongest fecundity compensation in hosts with an elevated stress response and the weakest compensation in hosts with a compromised stress response (Figure 3).

## 4 DISCUSSION

Fecundity compensation allows hosts to defend themselves against parasites by limiting fitness costs caused by parasite exposure (Heins & Baker, 2014; Minchella & Loverde, 1981; Schwanz, 2008; Thornhill et al., 1986; Vale & Little, 2012). Our results indicate that fecundity compensation has evolved and been conserved in the model host organism, *C. elegans*. We found that hosts exhibited greater fecundity compensation and enhanced their reproductive output if parasites were more harmful, thus highlighting the sensitivity of the life‐history shift. We also show that the fecundity compensation is linked to host general stress response and related innate immune system pathways triggered upon interactions with parasites.

Our results concur with previous findings indicating a role for generalized stress responses in other nonphysiological host responses (Schulenburg & Ewbank, 2007; Schulenburg & Müller, 2004). Contrary to expectations, nematodes with higher physiological innate immunity also show stronger behavioral avoidance responses (Schulenburg & Müller, 2004). These links have led to the hypothesis that behavioral avoidance mechanisms constitute part of a larger general stress response triggered upon exposure to parasites (Schulenburg & Ewbank, 2007). Thus, it is possible that increased fecundity compensation in *daf‐2* mutants and decreased fecundity compensation in *sek‐1* mutants are also a consequence of this general stress response. Indeed, the DAF‐2/DAF‐16 pathway is involved in the mitigation of physical evasion and reduced oral uptake of microbial parasites (Hasshoff, Böhnisch, Tonn, Hasert, & Schulenburg, 2007), in addition to the involvement in fecundity compensation that we report here, and is also critical to general stress responses in *C. elegans* (Evans, Chen, & Tan, 2008; Murphy et al., 2003). The p38 MAPK pathway is critical in *C. elegans* for defense against gram‐positive bacteria (Kim et al., 2002; Troemel et al., 2006) and, similarly to DAF‐2/DAF‐16, is also involved in general stress responses (Craig, Fink, Yagi, Ip, & Cagan, 2004). Furthermore, the p38 MAPK pathway is linked to the positive regulation of egg‐laying behavior in *C. elegans* (Kim et al., 2002), providing a mechanism by which it may mediate fecundity compensation. Interestingly, although hosts in the *Biomphalaria glabrata–Schistosoma mansoni* trematode system do not exhibit fecundity compensation during presumably stressful drought conditions, the parasites do ramp up offspring production (Gleichsner, Cleveland, & Minchella, 2016). Such a finding suggests that the connection between general stress and fecundity compensation can be complex in natural systems and that it can nevertheless be extended beyond hosts to parasites.

Trade‐offs are often predicted in evolutionary biology between indirect host responses and measures of innate immunity, a component of the generalized stress response (Schulenburg & Ewbank, 2007; Schulenburg, Kurtz, Moret, & Siva‐Jothy, 2009). The idea is that host resources should be invested into either direct responses, such as innate immunity, or indirect defenses, such as fecundity compensation, behavioral avoidance (Schulenburg et al., 2009), or antimicrobial secretions (Cotter & Kilner, 2010). However, evidence of such trade‐offs is scarce (Cotter, Littlefair, Grantham, & Kilner, 2013; Gupta et al., 2016; Lynch, Schlenke, & Roode, 2016), and our results might help to provide an explanation. We show that different host defenses to parasites may be mechanistically linked as hosts with a strengthened general stress response also show elevated fecundity compensation. This result, in combination with previous findings that behavioral avoidance responses and innate immunity are positively related (Schulenburg & Ewbank, 2007; Schulenburg & Müller, 2004), highlights that these host defense mechanisms are part of wider stress response upon parasite exposure and are thus not traded off.

Our experiments are based on two extreme phenotypes: nematodes with heightened immune response compared to nematodes with a suppressed immune response. Thus, to further explore the link between the general stress response and fecundity compensation, more experiments must be carried out along the phenotypic landscape.

## 5 CONCLUSIONS

Here, we have pinpointed a general mechanism by which fecundity compensation might operate. Future work is now needed to evaluate the specific roles of genes in the stress response pathways highlighted here. Such an approach will help us better understand the relationships between fecundity compensation and other host defenses that are triggered before and after exposure to parasites. In an evolutionary context, elucidating the mechanistic underpinning and complexity of fecundity compensation in *C. elegans* will ultimately yield a powerful system for directly testing how natural selection can shape host life‐history traits and other indirect defenses to parasites.

## CONFLICT OF INTEREST

The authors declare that they have no competing interests.

## AUTHORS' CONTRIBUTIONS

VLP, CR‐M, SAF, and KCK conceived and designed the study. VLP and SAF conducted the study. CR‐M and VLP conducted the statistical analysis. CR‐M, VLP, and KCK wrote the manuscript.

## ETHICS APPROVAL AND CONSENT TO PARTICIPATE

Not applicable.

## CONSENT FOR PUBLICATION

Not applicable.

## Data Availability

All data generated or analyzed during this study are available on the Dryad Digital Repository: DOI: https://doi.org/10.5061/dryad.198q4p4.
